# Comparative evaluation of shear bond strength of three flowable compomers on enamel of primary teeth: An *in-vitro* study

**DOI:** 10.4317/jced.52785

**Published:** 2016-07-01

**Authors:** Özge-Erken Güngör, Yıldırım Erdoğan, Ahmet Yalçın-Güngör, Hüseyin Alkış

**Affiliations:** 1 Assist. Prof. Dr., Akdeniz University, Faculty of Dentistry, Department of Paediatric Dentistry, Antalya, Turkiye; 2 Assist. Prof. Dr., Pamukkale University, Faculty of Dentistry, Department of Paediatric Dentistry, Denizli, Turkiye; 3 Assoc. Prof. Dr., Akdeniz University, Faculty of Dentistry, Department of Orthodontics, Antalya, Turkiye; 4 Assist. Prof. Dr., Suleyman Demirel University, Faculty of Dentistry, Department of Orthodontics, Isparta, Turkiye

## Abstract

**Background:**

The aim of the present study was to determine Shear bond strength (SBS) of different flowable compomers on the enamel surface of primary teeth. The null hypothesis to be tested was that none of the flowable compomer would differ significantly from the other two with respect to SBS. As a result, the tested materials that have the easiest application on child patient is preferred.

**Material and Methods:**

Sixty newly extracted non carious primary molars were selected. The buccal surface was cleaned and polished to obtain a flat enamel surface. The specimens were randomly divided into three groups of 20 teeth each, based on the flowable compomers applied, as follows: group I: Dyract Flow® (Dentsply, Konstanz, Germany); group II: Twinky Star Flow® (Voco, Cuxhaven, Germany); and group III: R&D Series Nova Compomer Flow® (Imicryl, Konya, Turkey).

**Results:**

SBS in group II (6.78± 0.45 MPa) were significantly lower than groups I and III (8.30 ± 0.29 and 8.43 ± 0.66 MPa, respectively) (*P*<.001). No significant difference was found between groups I and III (*P*<.05).

**Conclusions:**

Significant differences existed between the SBS of the groups. Therefore, the null hypothesis was rejected. Flowable compomers can provide adequate SBS with self-etching system at restoration of primary teeth. Thus, successful restorations in pediatric patients can be done in a practical way.

** Key words:**Flowable compomer, primary teeth, shear bond strength.

## Introduction

The researchers are trying to find that perfect restorative material to making the restorative process faster and more easily with acquisition of time and money and preventive of healthy tooth structure and also have highly adhesion to tooth structure in paediatric dentistry (1-3). Usually, glass ionomer cements and packable compomers are used for restorative treatment of primary teeth (4,5). These materials have both of advantages and disadvantages. Fluoride release is one of the important advantages of them, however, marginal leakage and less of bond strength are major problems still (6). In order to prevent this marginal leakage and reduce stress of under the restoration flowable compomers were developed recently (7).

Discovery of flowable compomers has been marked an era in paediatric restorative dentistry. Compared with packable compomers, flowable compomers show decreased anorganic filler content to improve viscosity and simplify application with increased elasticity. Flowable compomers have been claimed that they were the first choice for restorative treatment of class V and deep, narrow cavities, with difficult access angles of primary teeth especially under the difficult clinical conditions due to the cooperation problems of child patient by manufacturers. Also, they can use for class II cavities as liner because they have low elastic modulus that might be provided the material with stress absorbing ability and as pit and fissure sealant for permanent teeth (8). Especially in very young children they could apply into the cavity after minimal invasive approaches with lesser time and without the use of hand instruments.

The ability of flowable compomer to adhere to the enamel surface of the tooth affects the clinic success of treatment directly. The adhesion between dental material and teeth surface is obtained with different applications. Traditionally, etching of enamel surfaces with orthophosphoric acid, a concept first advised by Buonocore (9), and then 4th and 5th generation dental adhesives were defined, after etching procedure a bonding agent was used for strong adhesion. In recently ‘self-etch’ (6th generation) or ‘all-in-one’ (7th generation) adhesive systems have been developed with continuous technique improvements in dental adhesives (5,10). These systems do not need an “etch and rinse” application that is important for paediatric dentists because of shorter clinical application time and reduces technique sensitivity (11).

Several studies have evaluated the shear bond strength (SBS) of various restorative materials that were used to restoration of primary teeth (1,3,12-14) and only one study is available regarding the bond strengths of flowable compomers on permanent teeth (11). However, to the best of our knowledge, no studies have evaluated SBS of flowable compomers on the enamel surface of primary teeth.

The aim of the present study was to determine SBS of different flowable compomers on the enamel surface of primary teeth. The null hypothesis to be tested was that none of the flowable compomer would differ significantly from the other two with respect to SBS.

## Material and Methods

This study has been approved by Local Ethics Committee of Antalya Research Hospital. Sixty freshly-extracted, non-carious, primary molars without visible defects were used in this study. The reason of the teeth extraction is physiological root resorption of primary teeth and due to this phenomen becoming mobile. Following extraction, the teeth were cleaned mechanically to remove any residual tissue attached to the root surface. The teeth were washed under running tap water and stored in distilled water prior to the experiment. Each tooth was individually embedded in an auto-polymerizing acrylic resin (Meliodent; Heraeus Kulzer, Hanau, Germany). The buccal surfaces of the teeth were ground using pumice and then polished with silicon carbide paper to obtain a flat enamel surface under water-cooling. The teeth were rinsed completely with water and dried with compressed air. The teeth were randomly divided into three groups of 20 teeth each, based on the flowable compomers applied, as follows: group I: Dyract Flow® (Dentsply, Konstanz, Germany); group II: Twinky Star Flow® (Voco, Cuxhaven, Germany); and group III: R&D Series Nova Compomer Flow® (Imicryl, Konya, Turkey).

In group I, each tooth was etched with 37% phosphoric acid gel for 30 seconds. Then, all teeth were rinsed with water/spray combination for 30 seconds and dried until characteristic frosty white etched area is observed. Prime&Bond NT® (Dentsply, Konstanz, Germany) Adhesive and saturate apply to all surfaces for 20 seconds. Excess solvent removed by gently air drying for 5 seconds until Surfaces a uniform, glossy appearance achieved. Light cured for 10 seconds.

In groups II and III Futurabond U® (Voco, Cuxhaven, Germany) and R&D Series Nova Compobond® (Imicryl, Konya, Turkey) was applied respectively. With their microbrush, a thin uniform layer of sealant was applied on the enamel. To dry primer into a thin film, a gentle air burst was delivered.

A cylindrical polyethylene tube of standardized dimensions (3 mm in diameter and 3 mm in height) was placed on the enamel surface of each specimen. Flowable compomer was injected into the tube and polymerized according to manufacturers’ instructions using an LED curing unit (Elipar Free Light II; 3M/ESPE, St. Paul, MN, USA; light intensity: 1,000 mW/cm2). The flowable compomers were applied to each group.

Each specimen was placed in the Universal testing machine (Instron Universal test machine; Elista, Istanbul, Turkey), with the long axis of the specimen kept perpendicular to the direction of the applied force. The standard knife edge was positioned in the occlusocervical direction and in contact with the bonded specimen. Bond strength was determined in the shear mode at a cross-head speed of 0.5 mm/min until fracture occurred. The load at failure was recorded in Newtons (N) and converted into megapascals (MPa) by dividing the load at failure by the surface area of flowable compomer cylinders (mm2).

The Kolmogorov-Smirnov test was used to check the normality of the SBS distribution. The values indicated that the data were normally distributed (*p* = 0.794). Therefore, parametric tests were used. Descriptive statistics, including the mean, standard deviation (SD), and minimum and maximum values, were calculated for each of the groups tested. One-way ANOVA and Tukey’s multiple comparison tests were applied to assess the statistical significance of between-group differences. Significance for all statistical tests was predetermined at *p* < 0.05. All statistics were performed using SPSS Statistics 21.0 (SPSS Inc., Chicago, IL, USA).

## Results

This *in vitro* study was carried out to evaluate and compare the SBS of flowable compomers. Twenty samples from each group were tested for SBS and the values were recorded.

The mean score of Group 1 was 8.3 and SD was 0.29. The minimum score was 7.89 and the maximum score was 8.84.

The mean score of Group 2 was 6.78 and SD was 0.45. The minimum score was 6.04 and the maximum score was 7.58.

The mean score of Group 3 was 8.43 and SD was 0.66. The minimum score was 6.76 and the maximum score was 9.68.

**Figure 1:**
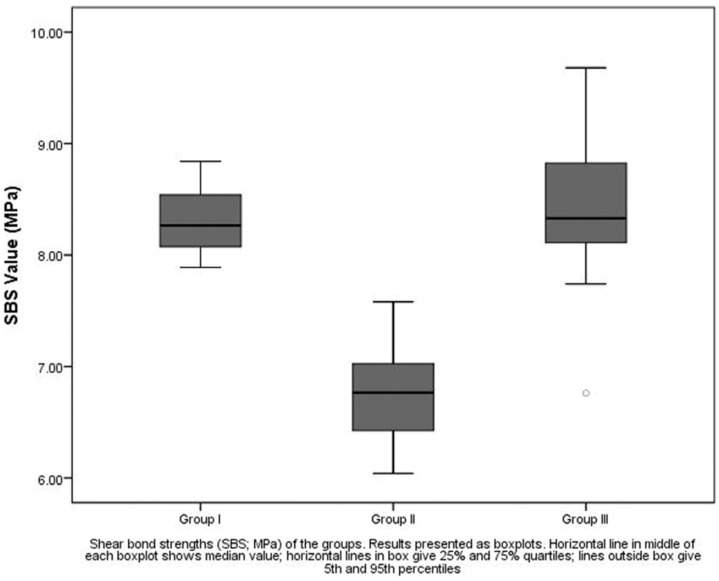
The descriptive statistics of the SBS (in MPa) of the groups are presented as boxplots.

The descriptive statistics of the SBS (in MPa) of the groups are presented as boxplots in figure 1. All groups showed clinically acceptable mean bond strengths. ANOVA indicated a significant difference between groups (*P*<.001) ( Table 1). Highest values of SBS were measured in group III (8.43 ± 0.66 MPa). SBS in group II (6.78± 0.45 MPa) were significantly lower than groups I and III (8.30 ± 0.29 and 8.43 ± 0.66 MPa, respectively) (*P*<.001). No significant difference was found between groups I and III (*P*<.05).

**Table 1:**
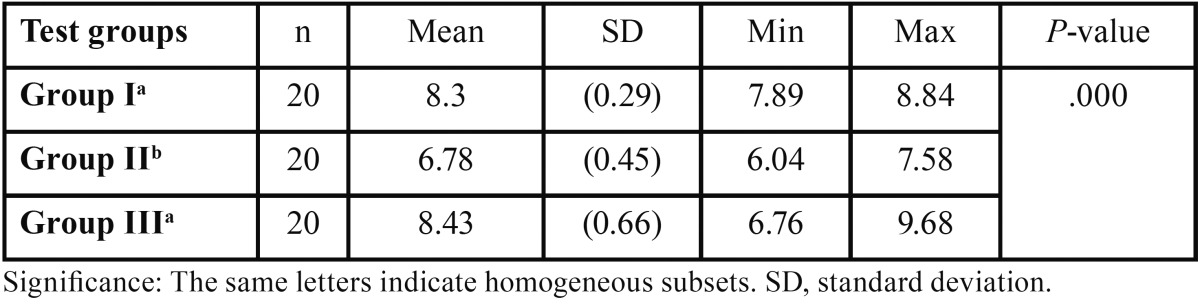
The results of the ANOVA comparing the shear bond strengths of the groups.

## Discussion

Packable compomers are commonly used for restoration of primary teeth. Although packable compomers have been shown to have a number of clinical advantages; there are some disadvantages while they were compared with flowable compomers. Flowable compomers can provide an opportunity of more conservative pit and fissure cavities of primary molars and simplified and fast restorative procedures. The morphology of pits and fissures facilitates accumulation of plaque and bacteria on the tooth surface. Due to this phenomenon the tooth has become caries susceptible (11). It was demonstrated that the beneficial effects of pit and fissure sealant to preventive caries not only permanent molars but also primary molars (13-15). Although it was known that flowable compomers was used as pit and fissure sealant both of permanent and primary teeth, only one study was made with flowable compomer at permanent teeth (11). Whatever the restorative material is used, all of them have a common objective that is strong bonding. Especially in paediatric dentistry, providing strong bonding between primary tooth and flowable compomers will make dental treatment easier.

Bond-strength tests are used to evaluate the bonding effectiveness of materials to tooth surface. The results of these laboratory tests are guide for clinical usage of materials (16). Therefore, it is important to make this kind of studies. However, there is no study evaluates the SBS of flowable compomers to enamel of primary teeth. So the aim of the present study was to evaluate the SBS of three different flowable compomers to enamel surface of primary molars. Although there was no statistically significant difference between Group I and Group III, Group II showed significantly lower SBS than those groups. Thus, the null hypothesis was rejected that there were no statistically significant differences in bond strength of different flowable compomers.

 Although different sizes of compomer blocks were used in various studies, the results are comparable to those of other studies, because the failure loads were recorded in Newton (N) and converted into MPa by dividing the failure load (N) by the surface area of compomer block (11,17). However we could not be able to compare our results with any similar study because this is the first study evaluates the SBS of flowable compomers applied to the primary tooth enamel.

In the present study, the results show that mean shear bond strength of Group I, II and III on primary teeth were 8.3 MPa, 6.78 MPa and 8.43 MPa respectively. Higher values were observed in a study done by Dhillon, *et al.* (11) and mean SBS of dyract flow on enamel surface of permanent teeth was 13.02 MPa with conventional etching. The difference could be due to difference of type of tooth used. Usually the adhesion between tooth surface and materials are weaker primary than to permanent tooth for both of enamel and dentin surface (11,18,19). The feasible reason for this difference could be attributed to the amount of mineral components in primary teeth and the differences of morphology and structure of primary teeth (20). Similar findings were reported when the same materials had been applied both of primary and permanent dentin surface the SBS of permanent teeth were found more high (3).

When the conventional etching and self-etching systems were compared, some studies were shown no statistically significant differences between these systems, one of these studies was performed on enamel surface of permanent teeth (13), and other was dentin surface of primary teeth (5). Conversely, it was reported in *in vitro* studies that SBS of pit and fissure sealants was higher with self etching primer as compared to conventional etch to enamel surface of permanent molars (11,21). In the present study flowable compomers were applied according to manufacturers’ instructions, therefore conventional etching system was used for group I and self-etching systems were used for other two groups. The reason of lower success of group II could be attributed to the differences in composition of these materials. It was reported that the bond strength of the unfilled resin sealant was found to be superior to that of the filled resin sealant in both of primary and permanent teeth (14). Colored compomers have been used in the restoration of primary teeth since 2002 (22), and Twinky star compomer is one them. This material is preferred especially by child patients because of its attractive colors. The amount of glitter particles were included in order to produce as a color effect and this situation may be the reason of the different values between the groups in our study. Although, in a clinical study, no significant difference was found among the conventional and colored compomers regarding marginal integrity, marginal discoloration, anatomic form, secondary caries and surface texture (23), further laboratory and clinical trial are required to evaluate flowable compomers.

Our results indicate that significant differences existed between the SBS of the groups. Therefore, the null hypothesis was rejected. Although Twinky star flow® yielded the lowest SBS values, R&D Series Nova Compomer Flow® which was applied with self-etching system as Twinky star flow® yielded the highest values. Flowable compomers can provide adequate SBS with self-etching system at restoration of primary teeth. Further studies on larger samples need to be undertaken using different flowable compomers with different surface treatments at both of primary and permanent teeth.
